# Roux-en-Y Intussusception: A Case Report

**DOI:** 10.7759/cureus.78088

**Published:** 2025-01-27

**Authors:** Lina Ibrahim, Santosh Potdar

**Affiliations:** 1 Chemistry, University of South Florida, Tampa, USA; 2 General Surgery, Tampa General Hospital Brooksville, Brooksville, USA; 3 General Surgery, Tampa General Hospital Spring Hill, Spring Hill, USA

**Keywords:** bariatric surgery complications, gastric bypass surgery, jejunal intussusception, roux-en-y, small bowel intussusception

## Abstract

Roux-en-Y gastric bypass (RYGB) is a bariatric surgical procedure commonly performed in adults to treat severe obesity. While RYGB is generally safe, it occasionally leads to rare but significant complications, including intussusception, a form of bowel obstruction caused by the invagination of an intestine segment from the proximal to the adjacent distal portion. We report a case of intussusception in a 74-year-old female patient who underwent RYGB 12 years prior. A computed tomography (CT) scan revealed jejunal intussusception accompanied by internal herniation, which was subsequently confirmed through exploratory laparotomy. The segments in intussusception were successfully reduced without any complications. Postoperative follow-up demonstrated the absence of recurrence or any additional complications.

## Introduction

Roux-en-Y gastric bypass (RYGB) is a bariatric surgical procedure effective for people with severe obesity in sustaining weight loss. It is the recommended bariatric procedure due to its promising benefit-to-risk ratio, and is most common amongst women; about 85% of RYGB patients are women and 15% male, ranging between the ages of 20-67 years [1]. Over the years 2014-2018, 38% of gastric bypass procedures were found to be RYGB [1]. This procedure involves transversely cutting the stomach and creating a smaller pouch of approximately 1 ounce capacity [2]. The newly formed upper pouch is attached to the proximal jejunum, bypassing the remaining of the stomach, the duodenum, and a fraction of the proximal jejunum, thus creating the Roux limb [3,4]. The bypassed segments, which contain the essential digestive enzymes, form the biliopancreatic limb and are rejoined from the duodenum to the distal segment of the jejunum [4]. Intussusception is a rare complication that can potentially follow the procedure, involving the invagination, or telescoping, of the intestine into itself from the proximal segment into the adjacent distal segment [5]. Its occurrence is especially rare in adults, with studies suggesting approximately 0.1%-0.3% of gastric bypass patients, and roughly 0.64% of RYGB patients, develop intussusception [5,6]. While the etiology is still unknown, common clinical symptoms include diarrhea, bilious vomiting, abdominal pain, abdominal mass, abdominal distention/swelling, and currant jelly stool [7,8].

## Case presentation

**Figure 1:**
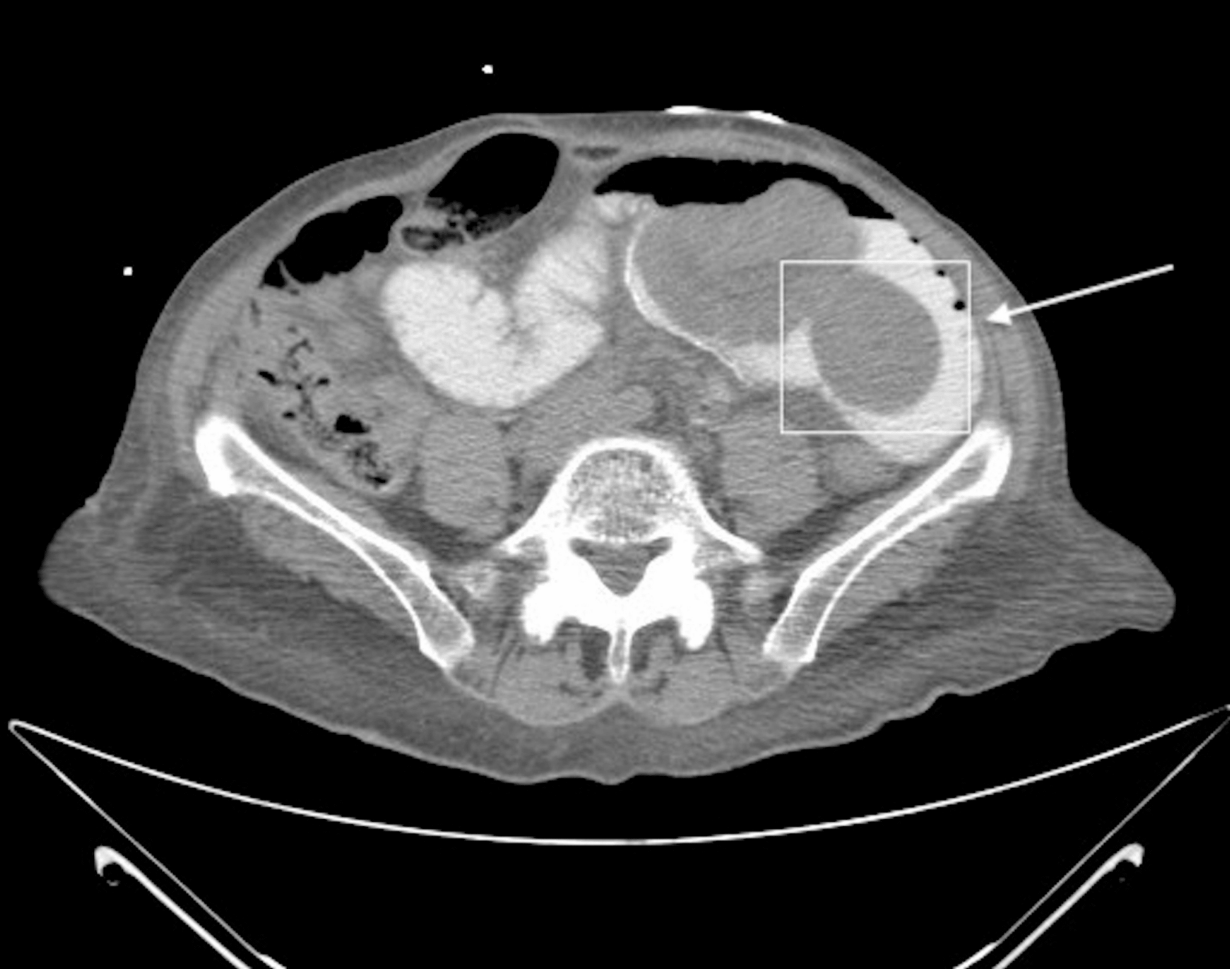
CT scan shows dilated small bowel loops involving left upper quadrant jejunal loops due to the intussusception CT: computed tomography

A 74-year-old female patient was admitted to Tampa General Hospital through the emergency department. She reported that her symptoms began with moderate colicky-natured abdominal pain, followed by abdominal distention, and then dark stool. The duration of the symptoms was two days prior to the emergency room admission. Physical examination revealed no pathologic conditions of the mesentery nor the retroperitoneal lymph nodes, as well as no free air or fluid in the peritoneum, and no hernia or mass in the abdominal wall. Her vitals appeared normal, and laboratory investigations were all normal except for an increase in white blood cell (WBC) count, indicative of a potential infection. Her medical history included an RYGB with the Roux limb in antecolic position 12 years prior and a cesarean delivery. The initial consult was for a suspected small bowel obstruction; however, a computed tomography (CT) scan with oral contrast revealed a dilated loop of the small bowel in the left upper quadrant, suggestive of intussusception involving the proximal jejunum telescoping into the ileum (Figure 1). The CT also revealed internal herniation within the enteroenteric anastomosis of the jejunum and mild swirling of the mesentery. There were no observations of any perforation or drainable fluid nor a hernia or mass in the abdominal wall.

**Figure 2:**
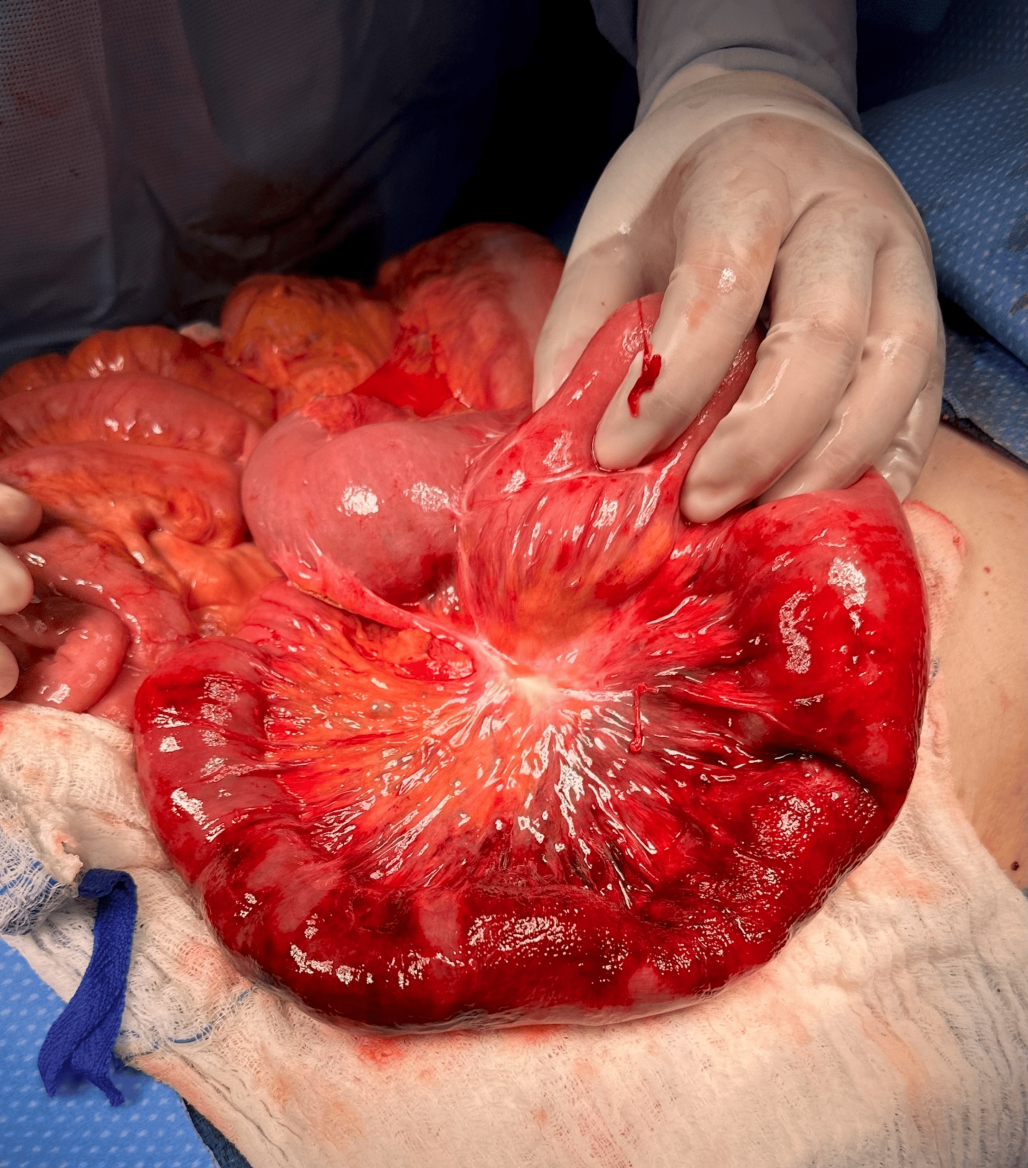
Exploratory laparotomy showed approximately a one-foot segment of the jejunum (intussusceptum) telescoping into the ileum (intussusceptien)

**Figure 3:**
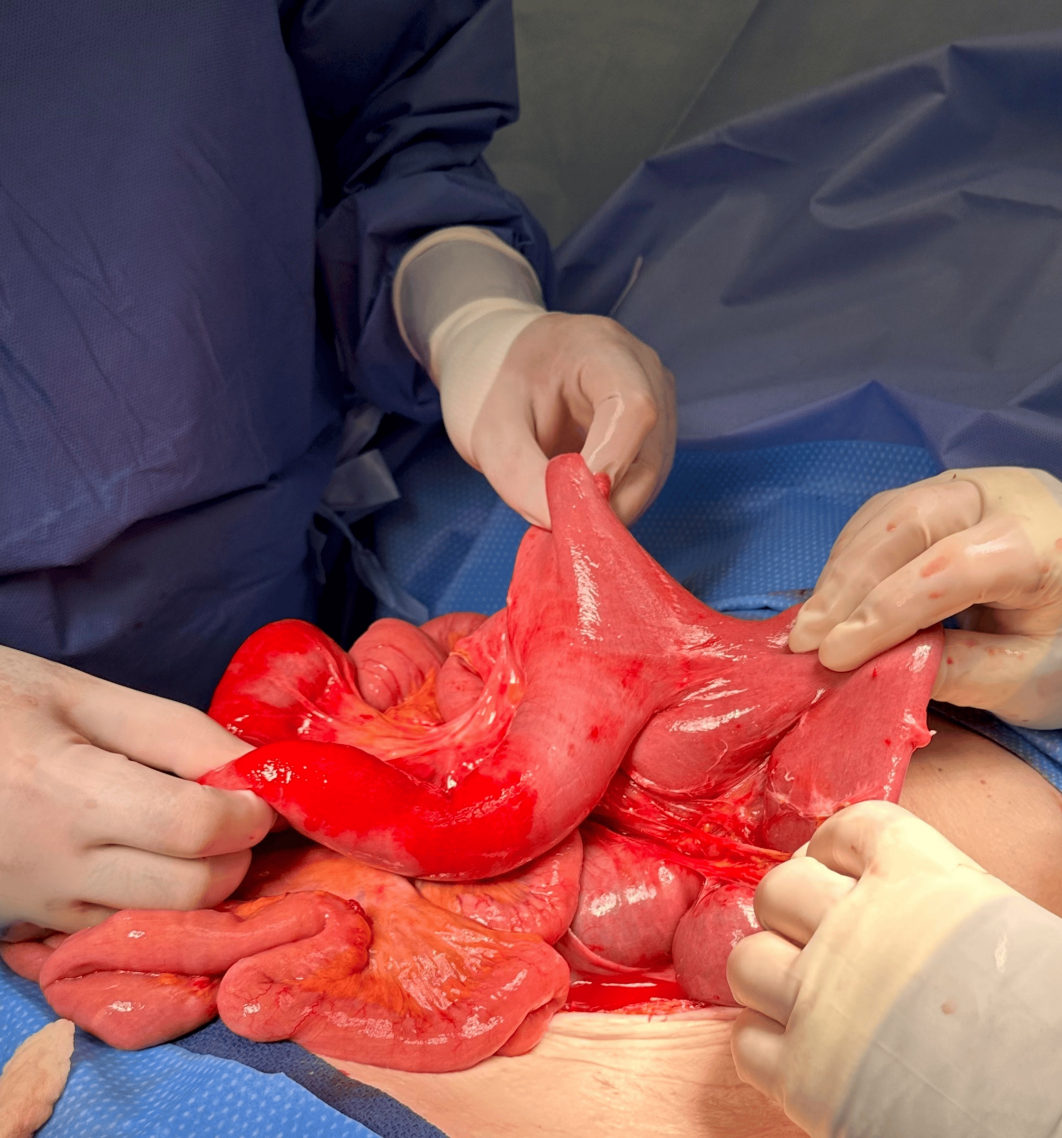
Segment of the small bowel in intussusception was reduced and appeared a pink color after warming. No signs of ischemia or perforation

An exploratory laparotomy was performed, and a very large enteroenteric anastomotic site from the previous surgery was found, containing a large loop of the ascending jejunum (Figure 2). Approximately one foot of the small bowel was involved in intussusception and was reduced carefully and successfully with no perforation or ischemia (Figure 3). No sign of any lead point was identified. The abdomen was then closed without any complications. Five days following the surgical procedure, a CT scan administered with oral contrast revealed normal findings. The patient was able to tolerate a gastrointestinal soft diet and was then discharged. She was prescribed a five-day course of antibiotics for possible infection in the intestine. Nine-month postoperative follow-up showed no complications following the reduction. 

## Discussion

Intussusception in adults, although uncommon, is a potential complication following RYGB, and may pose significant clinical challenges. Reports of intussusception tend to be most prevalent in infancy or early childhood, characterized by palpable mass or visible protrusion through the anus, whereas in adults, the diagnosis is often delayed or misdiagnosed due to the lack of distinct symptoms [8]. In adults, intussusception accounts for 5%-10% of intestinal obstructions diagnosed and can be attributed to pathological abnormalities including Meckel’s diverticulum, neoplasm, malignancies, and benign polyps. [9,10]. However, these lead points are not well-documented in RYGB patients [11]. The mechanism causing intussusception in RYGB patients remains poorly understood, with theories of the jejuno-jejunal (j-j) anastomosis acting as a lead point or of the Roux limb causing the peristaltic movement to become asynchronous which leads to the bowel to invaginate into itself. [12]. In this case, it is plausible that the gastric bypass procedure had unintended consequences that contributed to the development of intussusception. The creation of the antecolic Roux limb potentially caused a disruption in the peristalsis leading to dysmotility and susceptibility to intussusception. The patient's symptoms closely aligned with those reported of intussusception, specifically abdominal pain, abdominal distention, and dark stool. If left untreated, intussusception can potentially lead to life-threatening complications such as peritonitis, ischemia, necrosis, or perforation [13]. 

A meta-analysis conducted by Oor et al. in 2021 of RYGB intussusception included 74 published studies from 1991 to 2020, revealing 191 RYGB patients that later developed intussusception and a collective incidence of 0.64% following RYGB [6]. A total of 98% of the patients were female, and the patients' ages ranged between 22 and 60 years. The interval between RYGB and intussusception ranged between six months and 38 years, and the interval between treatment and recurrence ranged between two weeks and 40 months. Antecolic Roux limbs were slightly more seen in intussusception incidence following RYGB than retrocolic Roux limbs, with incidence rates of 50% and 47%, respectively. Literature regarding intussusception and Roux limb positioning is not yet definitive; however, most of the included studies showed a higher percentage of intussusception involving antecolic Roux limbs. Of the 34% who underwent a resection of the segment, 22% reported a recurrence in intussusception. The literature available regarding the treatment of RYGB intussusception differs. Some strictly recommend resection due to the inability to detect a lead point, while others suggest reduction due to its noninvasive nature, lower risk of postsurgical complications, bowel preservation, no risk of anastomotic leakage, and reduced hospital length of stay, only if there is no ischemia and intussusception appears reducible [5,14]. The largest study in this analysis reported a 25% recurrence rate following reduction and 12.5% following resection; however, the rates vary across various studies. Aside from reduction and resection, postoperative complication rates in different studies reported range between 4% and 29%, and complications including the need for blood transfusion, infection, and small bowel obstruction.

The clinical presentations of intussusception can vary widely, necessitating a prompt diagnosis and elevated clinical suspicion. Imaging techniques serve as valuable diagnostic tools to identify the condition; a CT scan was utilized for this case, due to its high accuracy in detecting small bowel obstruction, with oral contrast to determine surgical necessity [15]. The scan revealed markedly dilated small bowel loops involving left upper quadrant jejunal loops, consistent with intussusception. A substantial portion of the intestine herniated within the entero-enteric anastomosis, causing upstream dilatation and small bowel obstruction. The contrast did not pass beyond this point. No indications of gangrene were observed during the exploratory laparotomy, deeming resection unnecessary and reduction as the more appropriate approach. Although specific guidelines for intussusception surveillance are limited, the lack of lead point identification and reported recurrence rates suggest a need for structured, long-term postoperative monitoring; given that recurrences can occur months or even years after the initial event, follow-up care strategies should be carefully designed to monitor for this condition over time [16].

## Conclusions

Intussusception in adults is a rare but clinically significant complication following RYGB, necessitating timely recognition and intervention. The successful management of this patient without resection of the segment in intussusception highlights the importance of prompt surgical exploration and accurate imaging. Long-term postoperative follow-ups are essential to monitor potential recurrence and strengthen clinical awareness to reduce associated risks.
